# Analysis of *Saprolegnia parasitica* Transcriptome following Treatment with Copper Sulfate

**DOI:** 10.1371/journal.pone.0147445

**Published:** 2016-02-19

**Authors:** Kun Hu, Rong-Rong Ma, Jun-Ming Cheng, Xin Ye, Qi Sun, Hai-Lan Yuan, Nan Liang, Wen-Hong Fang, Hao-Ran Li, Xian-Le Yang

**Affiliations:** 1 National Pathogen Collection Center for Aquatic Animals, Shanghai Ocean University, 999 Hucheng Huan Road, Shanghai 201306, China; 2 East China Sea Fisheries Research Institute Chinese Academy of Fishery Sciences, Shanghai 201306, PR China; University of California-Riverside, UNITED STATES

## Abstract

**Background:**

Massive infection caused by oomycete fungus *Saprolegnia parasitica* is detrimental to freshwater fish. Recently, we showed that copper sulfate demonstrated good efficacy for controlling *S*. *parasitica* infection in grass carp. In this study, we investigated the mechanism of inhibition of *S*. *parasitica* growth by copper sulfate by analyzing the transcriptome of copper sulfate—treated *S*. *parasitica*. To examine the mechanism of copper sulfate inhibiting *S*. *parasitica*, we utilized RNA-seq technology to compare differential gene expression in *S*. *parasitica* treated with or without copper sulfate.

**Results:**

The total mapped rates of the reads with the reference genome were 90.50% in the control group and 73.50% in the experimental group. In the control group, annotated splice junctions, partial novel splice junctions and complete novel splice junctions were about 83%, 3% and 14%, respectively. In the treatment group, the corresponding values were about 75%, 6% and 19%. Following copper sulfate treatment, a total 310 genes were markedly upregulated and 556 genes were markedly downregulated in *S*. *parasitic*a. Material metabolism related GO terms including cofactor binding (33 genes), 1,3-beta-D-glucan synthase complex (4 genes), carboxylic acid metabolic process (40 genes) were the most significantly enriched. KEGG pathway analysis also determined that the metabolism-related biological pathways were significantly enriched, including the metabolic pathways (98 genes), biosynthesis of secondary metabolites pathways (42 genes), fatty acid metabolism (13 genes), phenylalanine metabolism (7 genes), starch and sucrose metabolism pathway (12 genes). The qRT-PCR results were largely consistent with the RNA-Seq results.

**Conclusion:**

Our results indicate that copper sulfate inhibits *S*. *parasitica* growth by affecting multiple biological functions, including protein synthesis, energy biogenesis, and metabolism.

## Introduction

*Saprolegnia parasitica*, a destructive oomycete pathogen that infects fish and fish eggs, is detrimental to freshwater hatcheries [1, 2]. It often causes a severe disease, characterized by visible white or grey patches of filamentous mycelium on the freshwater fish or their eggs, which seriously affect their value, and can result in significant economic loss [3]. *S*. *parasitica* infection in aquaculture is prevalent, particularly under stress conditions such as rapid changing of temperatures or water quality [3]. The infection of *S*. *parasitica* can be controlled with effective substances. However, malachite green, an organic dye that is very efficient at controlling *S*. *parasitica* infection [4], was banned worldwide in 2002 [3] because of its carcinogenic and toxicological effects on animal health [5, 6]. Therefore, it is imperative to find an alternative.

Humic substances with higher molecular weights and aromaticity contain a high number of organic radicals that are efficient for reducing fungal growth. Humic substance development of internal oxidative stress could be the mechanism for the observed inhibition of *S*. *parasitica* growth [7]. Clotrimazole is another potent treatment agent of *S*. *parasitica* infection by inhibiting sterol 14 alpha-demethylase (CYP51)[8]. Meanwhile, Saprolmycin A-E are reported to have highly effective anti–*S*. *parasitica* selective activity as well [9]. Recently, our lab found copper sulfate could control *S*. *parasitica* infection in grass carp with fairly good efficacy, which suggested that copper sulfate might be used as a drug additive to control *S*. *parasitica*infection in the aquaculture industry [10]. However, the mechanism of inhibition of *S*. *parasitica* growth by copper sulfate is unclear and it is necessary to study.

In this study, we performed transcriptome analysis to investigate the mechanism of copper sulfate inhibition of *S*. *parasitica* growth. We found that copper sulfate might inhibit protein synthesis, energy biogenesis, and metabolism in *S*. *parasitica*. Our findings may describe the mechanism of action of copper sulfate for controlling *S*. *parasitica* growth in water.

## Materials and Methods

The experimental protocol was established, according to the ethical guidelines of the Helsinki Declaration and was approved by the Human Ethics Committee of Shanghai Ocean University, China.

### Maintenance and treatment of *S*. *parasitica*

*Saprolegnia parasitica* (ATCC 200013) was obtained from American Type Culture Collection (Manassas, USA) and cultured on potato dextrose agar (Sinopharm Chemical Reagent Co., Ltd., Shanghai, China) for 4 days at 20°C. A mold colony, composed of the countless *S*. *parasitica*, was treated with copper sulfate (0.5 mg/L, Shanghai Guoyao Chemical Reagent Co. Shanghai, China) for 30 min. Additional *S*. *parasitica* were treated with sterile H_2_O as a control. Afterward, he *S*. *parasitica* were collected by centrifugation and stored in liquid nitrogen for further experiment.

### RNA isolation, RNA sequencing (RNA-seq) library construction and sequencing

We extracted total RNA from samples using TRIzol reagent (Invitrogen, USA) according to the manufacturer’s instruction. We removed DNA contaminants after treatment with RNase-free DNase I (Takara Biotechnology, Dalian, China), and confirmed RNA integrity with a minimum RNA integrated number value of 8 by an Agilent 2100 Bioanalyzer (Agilent, Santa Clara, USA). We isolated poly(A) mRNA using oligo-dT beads and then treated it with fragmentation buffer. We then transcribed the cleaved RNA fragments into first-strand complementary DNA (cDNA) using reverse transcriptase and random hexamer primers, followed by second-strand cDNA synthesis using DNA polymerase I and RNase H. The double-stranded cDNA was end-repaired using T4 DNA polymerase, Klenow fragment, and T4 polynucleotide kinase, followed by the addition of a single (A) base using Klenow 39–59 exo2 polymerase, and ligation with an adapter or index adapter using T4 quick DNA ligase. We selected adaptor-ligated fragments according to their size on agarose gel. The desired cDNA fragment size range was excised from the gel, and PCR was performed to selectively enrich and amplify the fragments. Following validation on an Agilent 2100 Bioanalyzer and ABI StepOnePlus Real-Time PCR System (ABI, California, USA), the cDNA library was sequenced on a flow cell using high-throughput 101-bp pair-end mode on an Illumina HiSeq 2500 (Illumina, San Diego, USA) unit.

### Sequencing, data processing, and quality control

We filtered low-quality reads and removed 3′ adapter sequences using Trim Galore. The obtained reads were cleaned using FastQC software (http://www.bioinformatics.babraham.ac.uk/projects/fastqc/), and we evaluated the content and quality of the nucleotide bases in the sequencing data. Next, we conducted a comparative analysis with the reference genome (ASM15154v2). For each sample, sequence alignment with the reference genome sequences was carried out using Tophat [11].

### Identification of differential expression genes

To investigate the expression level of each transcript of the two different treatment groups, we used Cuffnorm, a program to estimate the expression level (relative abundance) of a specific transcript expressed using FRKM (Fragments per kilobase of transcript per million fragments mapped) [12]. Generally, when FPKM > 0.1, this indicates that a given transcript is expressed. The expression level of each transcript was transformed using base 2 log_2_(FPKM+1). Meanwhile, we used the Cuffdiff program to calculate the fold change of a transcript and to screen all differentially expressed genes (DEGs). Two-fold changes with p-values < 0.05 were considered significant.

### GO functional annotation and enrichment analysis for differentially expressed genes

Initially, DEGs were annotated against the UniProt database (http://www.uniprot.org/). Next, we analyzed the functional annotation by gene ontology terms (GO; http://www.geneontology.org) with Blast 2GO (https://www.blast2go.com/) [13].

All DEGs were mapped to GO terms in the GO database, and gene numbers were calculated for every term, followed by an ultra-geometric test to find significantly enriched GO terms in DEGs compared to the transcriptome background. The formula used was as follows:

$$P=1-\sum_{i=0}^{m-1}\frac{\left(\begin{array}{l} M\\ i \end{array}\right)\left(\begin{array}{l} N-M\\ n-i \end{array}\right)}{\left(\begin{array}{l} N\\ n \end{array}\right)}$$

Where N is the number of all genes with GO annotation; n is the number of DEGs in N; M is the number of all genes annotated to specific GO terms; m is the number of DEGs in M. The calculated p-value was subjected to Bonferroni correction. A corrected p-value < 0.05 was defined as a threshold, and then GO terms were considered significantly enriched in the DEGs.

### DEGs pathway analysis

DEGs were annotated to the KEGG pathways database using the online KEGG Automatic Annotation Server (KAAS, available online: http://www.genome.jp/keg/kaas/). Enriched DEG pathways were identified using the same formula as that in GO analysis. In KEGG pathway analysis, N is the number of all genes with KEGG annotation, n is the number of DEGs in N, M is the number of all genes annotated to specific pathways, and m is the number of DEGs in M.

### DEG verification using qRT-PCR

The expression levels of DEGs identified in the RNA-seq analysis were verified with quantitative RT-PCR (qRT-PCR). Primers were designed using the Primer 5.0 software(Premier company, Canada), and SpTub-b (*S*.*parasitica* Tub-b)was used as the reference gene [14, 15].

The reactions were performed in a 25 μl volume, composed of 2μl cDNA, 0.5μl of both the forward and reverse primer (10μM), 12.5μl SYBR Premix Ex Taq (2×) and 9.5μl RNase-free H_2_O. The thermal cycling program was 95°C for 30 s, followed by 40 cycles of 95°C for 5 s, 60°C for 30s, and 72°C for 30 s. Melting curve analysis was performed at the end of the qRT-PCR cycles to confirm the PCR specificity. Three replications were carried out and analysis of relative gene expression data using 2^−△△CT^ Method [16].

## Results

### Illumina sequencing and quality assessment

We performed RNA-seq using the Illumina sequencing platform to examine the effect of copper sulfate on the *S*. *parasitica* transcriptome. After filtering and quality checks of the raw reads (47,614,574 and 51,163,206 for the control and treatment groups, respectively), there were approximately 44 million (44,676,046) and 45 million (45,325,202) trimmed reads with trim rates of 95.0% and 88.6% for control and the treatment samples, respectively. Meanwhile, the respective average length of reads was 94.5 and 93.8 bp (Table 1), indicating successful sequencing of the *S*. *parasitica* transcriptome. And trimmed reads were used for the subsequent analysis.

**Table 1:** Summary of reads in *S*. *parasitica* transcriptome sequencing.

Sample	Raw reads	Trimmed reads	Average length	Trim rate
Control	47,614,574	44,676,046	93.8 bp	95.0%
Copper sulfate	51,163,206	45,325,202	94.5 bp	88.6%

### Comparative analysis with reference genome

The trimmed reads of *S*. *parasitica* transcriptome were compared with the reference genome sequence. The total mapped rates of the reads with the reference genome were 90.50% in the control group and 73.50% in the experimental group. Uniquely mapped reads were about 14 million (14,952,142) for the control and 29 million (29,202,690) for the experimental group, and accounted for 33.47% and 64.42% of total reads, respectively. Multiple mapped reads were about 25 million (25,476,517) for the control group and 4 million (4,116,175) of the experimental group, and make up 57.03% and 9.08% of total reads, respectively. Total unmapped reads accounted for 9.50% in the control group and 26.50% in the experimental group (Table 2).

**Table 2:** Statistical results of trimmed reads mapping with reference genome.

Map to genome	Control		Copper sulfate	
	Reads numbers	percentage	Reads numbers	percentage
Total reads	44,676,046	100.00%	45,325,202	100.00%
Total mapped	40,428,659	90.50%	33,318,865	73.50%
Uniquely mapped	14,952,142	33.47%	29,202,690	64.42%
Multiple mapped	25,476,517	57.03%	4,116,175	9.08%
Total unmapped	4,247,387	9.50%	12,006,337	26.50%

**Fig 1:**
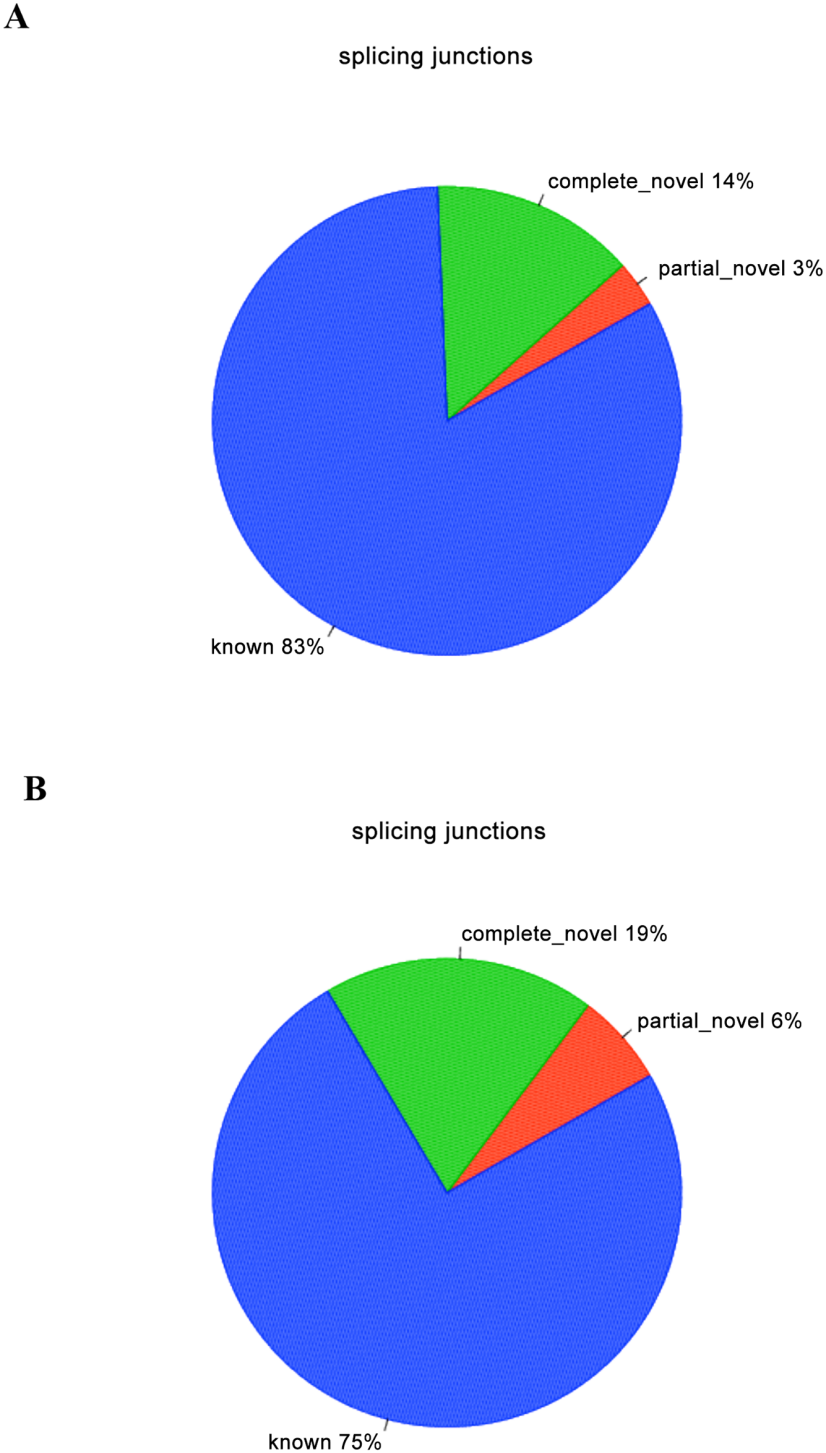
Types of splice junctions in the *S*. *parasitica* transcriptome in the control (Fig 1A) and experimental group (Fig 1B) as compared to the reference genome. Pie charts break down the known, partial novel and novel splice junctions identified in this study. Known splice junctions: both ends of splice junction belong to the annotated splice junction in the known genetic model; partial novel splice junctions: one end of splice junction belongs to the annotated splice junction in the known genetic model and the other end belongs to the new splice junction; complete novel splice junctions:both ends of splice junction belong to the new splice junction.

Additionally, splice junctions were discovered using TopHat, which can identify splice variants of genes. In the control group, known splice junctions accounted for 83% of total splice junctions annotated in the reference genome sequence. Partial novel splice junctions were about 3% of total splice junctions, and complete novel splice junctions were about 14% of total splice junctions (Fig 1A). In the experimental group, known splice junctions, partial novel splice junctions and complete novel splice junctions were about 75%, 6% and 19%, respectively (Fig 1B). These results indicate that the transcriptome of *S*. *parasitica*treated by copper sulfate contained more partial novel and complete novel splice junctions than the *S*. *parasitica* of control group.

### Analysis of DEGs

**Fig 2:**
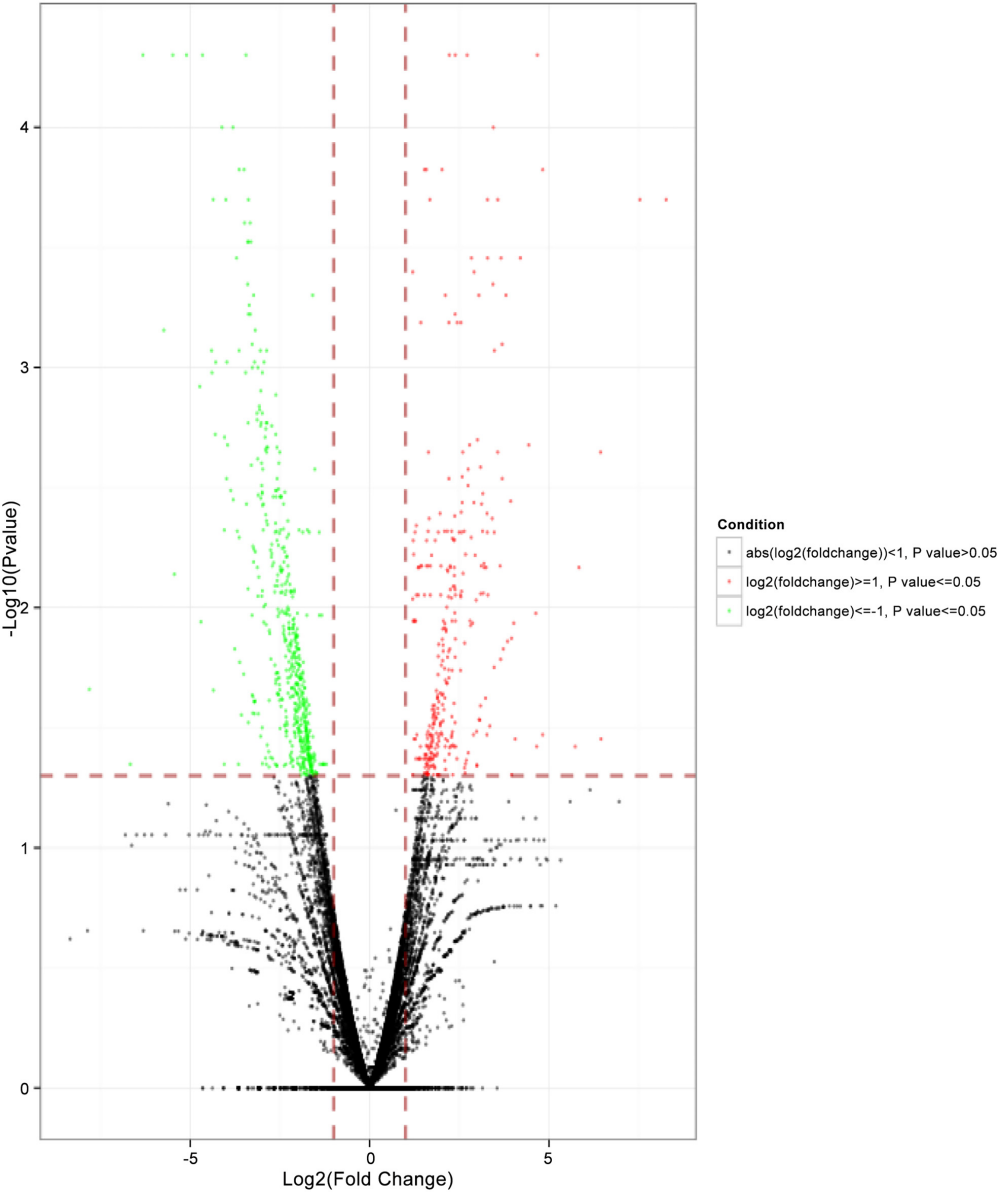
Effect of copper sulfate treatment on the gene expression profile in *S*. *parasitica* gene expression profile following copper sulfate treatment. Volcanic plot of the degree of differences in the expression profile of *S*. *parazitica* after treatment with copper sulfate. X-axis, log_2_(fold change); Y-axis,-log_10_(Pvalue). Red, the significantly upregulated genes, green, the significantly downregulated genes. Each dot represents one gene.

**Fig 3:**
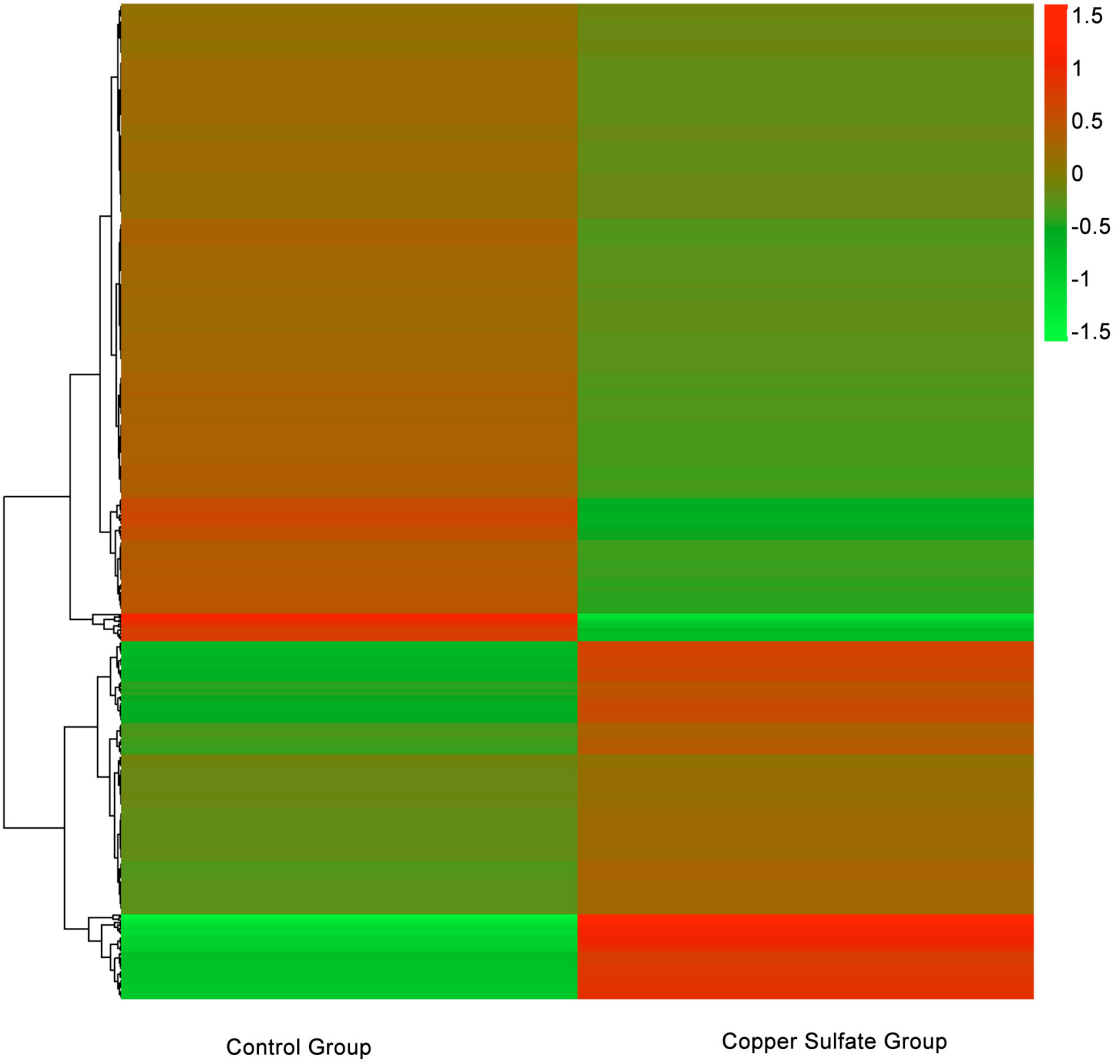
Effect of copper sulfate treatment on gene expression profile using pattern clustering. Red, upregulated genes; green, downregulated genes. Each line represents one gene.

To identify DEGs in *S*. *parasitica* following copper sulfate treatment, we used Cuffdiff program to generate *S*. *parasitica* gene expression profiles (Figs 2 and 3). The program identified 310 genes were markedly upregulated and 556 genes were markedly down regulated in *S*. *parasitica* following copper sulfate treatment, which indicates that copper sulfate affects *S*. *parasitica* gene expression.

### GO annotation of DEGs

To investigate the biological functions in which DEGs are involved in *S*. *parasitica*, we classified the DEGs according to GO classification following copper sulfate treatment in *S*. *parasitica*. This analysis identifies the main biological functions DEGs exercise. The GO functional enrichment analysis also involved cluster analysis of expression patterns. Thus, the expression patterns of DEGs annotated with a given GO term were easily obtained. All annotated genes were classified into three GO domains: biological processes, cellular component, and molecular function. Dissimilar expression profiles were obtained from the DEGs in the treated and control samples, revealing the obvious effect of copper sulfate on *S*. *parasitica* metabolism and physiology. The expression profiles of the three GO domains were as follows (each domain showed the top 10 terms).

**Fig 4:**
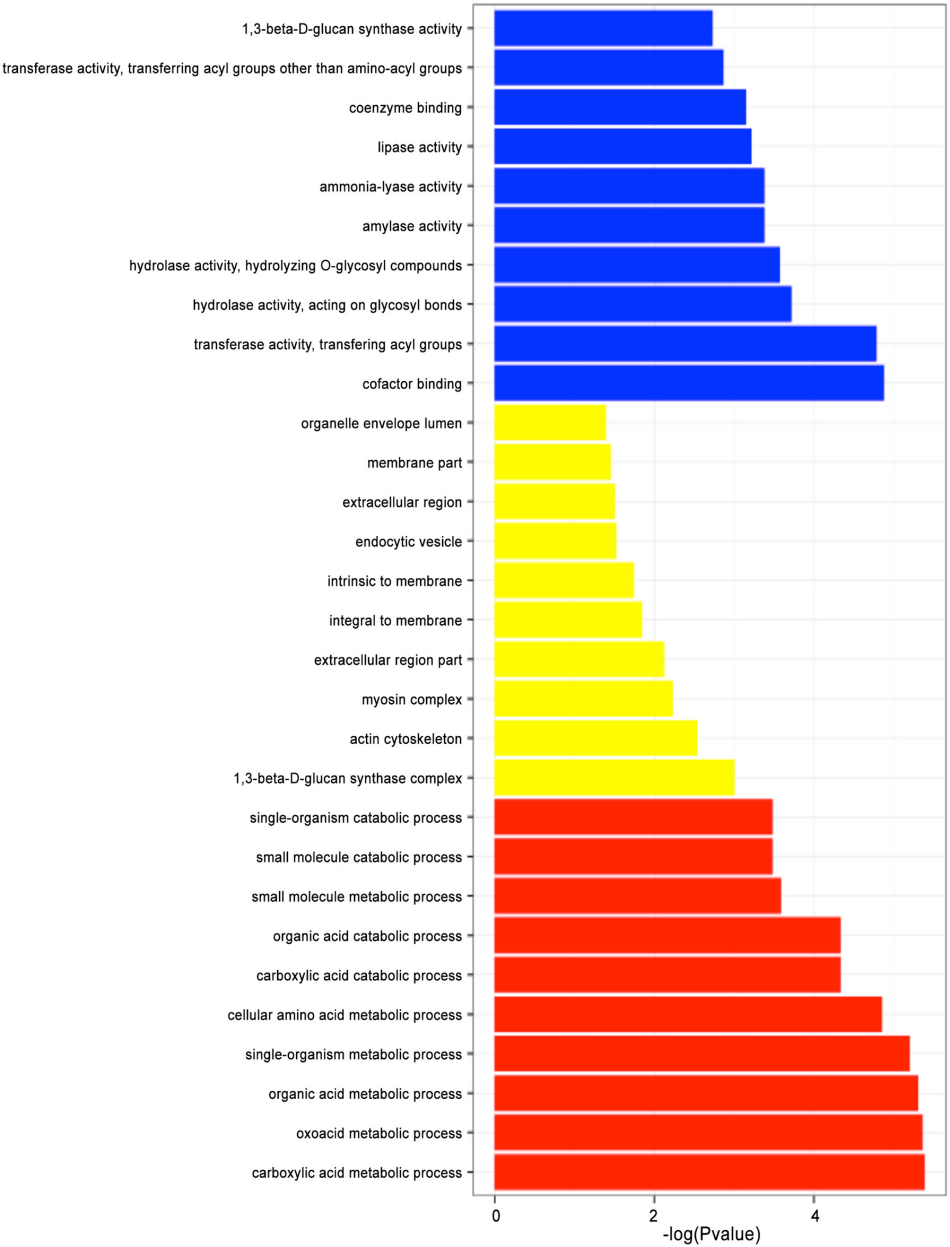
Histogram representation of enriched category of GO annotation of DEGs in *S*. *parasitica* following copper sulfate treatment. GO categories (y-axis) were grouped into three main ontologies: biological process, cellular component, and molecular function. The x-axis indicates the statistical significance of the enrichment. All annotated genes were classified into three GO domains: biological processes, cellular component, and molecular function. Red histogram represents the biological processes, the yellow histogram represents cellular component, and the blue histogram represents molecular function. Dissimilar expression profiles were obtained from DEGs in the treated and control samples, revealing the obvious effect of copper sulfate on *S*. *parasitica* metabolism and physiology.

Molecular function: 1,3-beta-D-glucan synthase activity (4 genes), transferase activity, transferring acyl groups other than amino-acyl groups (16 genes), coenzyme binding (22 genes), lipase activity (9 genes), ammonia-lyase activity (3 genes), amylase activity (3 genes), hydrolase activity, hydrolyzing O-glycosyl compounds (20 genes), hydrolase activity, acting on glycosyl bonds (21 genes), transferase activity, transferring acyl groups (25 genes), cofactor binding (33 genes) (Fig 4).

Cellular component: organelle envelope lumen (1 gene), membrane part (65 genes), extracellular region (9 genes), endocytic vesicle (2 genes), intrinsic to membrane (62 genes), integral to membrane (62 genes), extracellular region part (4 genes), myosin complex (9 genes), actin cytoskeleton (11 genes), 1,3-beta-D-glucan synthase complex (4 genes) (Fig 4).

Biological processes: single-organism catabolic process (9 genes), small molecule catabolic process (9 genes), small molecule metabolic process (90 genes), organic acid catabolic process (9 genes), carboxylic acid catabolic process (9 genes), cellular amino acid metabolic process (32 genes), single-organism metabolic process (141 genes), organic acid metabolic process (40 genes), oxoacid metabolic process (40 genes), carboxylic acid metabolic process (40 genes) (Fig 4).

Cofactor binding (33 genes), 1,3-beta-D-glucan synthase complex (4 genes), carboxylic acid metabolic process (40 genes) were the most significant GO terms. Globally, the 1515 GO terms were annotated, and the 262 GO terms underwent dramatic expression changes.

Following copper sulfate treatment, genes related to molecular function, including SPRG_10490(ornithine-oxo-acid transaminase), SPRG_03427 (oxoglutarate dehydrogenase), SPRG_06771 (tyrosine aminotransferase), SPRG_15406 (trehalase), and biological process related genes such as SPRG_11730 (histidine ammonia-lyase), SPRG_05010 (tryptophanyl-tRNA synthetase), SPRG_01675 (homogentisate 1%2C2-dioxygenase), SPRG_11441 (uridine phosphorylase), SPRG_11440 (uridine phosphorylase) were repressed, while molecular function related genes like SPRG_07378 (threonine ammonia-lyase) and biological process related genes like SPRG_01336 (maleylacetoacetate isomerase), SPRG_00691 (imidazoleglycerol-phosphate dehydratase) were upregulated. Most of these genes are responsible for protein synthesis, suggesting that copper sulfate mostly affects protein synthesis in *S*. *parasitica*. The expression of genes related to cellular flow, signal transduction, and cellular transport was also altered in S. *parasitica* after treatment with copper sulfate (including downregulated genes such as SPRG_05020, SPRG_13728, SPRG_03925, SPRG_07166, SPRG_17366, SPRG_20259 and SPRG_00668, and upregulated genes, such as SPRG_19069, SPRG_06201 and SPRG_20593), which are closely linked with substance metabolism. Additionally, copper sulfate treatment specifically activated SPRG_08152 (hypothetical protein), SPRG_09968 (hypothetical protein) and SPRG_06695 (hypothetical protein), which only are only expressed in the copper sulfate treated samples. The functions of hypothetical proteins were not clear, but were closely related to the biological functions of organism.

Overall, the above analyses indicate that copper sulfate inhibits *S*. *parasitica* growth by affecting multiple biological functions, including protein synthesis and metabolism.

### KEGG pathway analysis of differentially expressed genes

**Fig 5:**
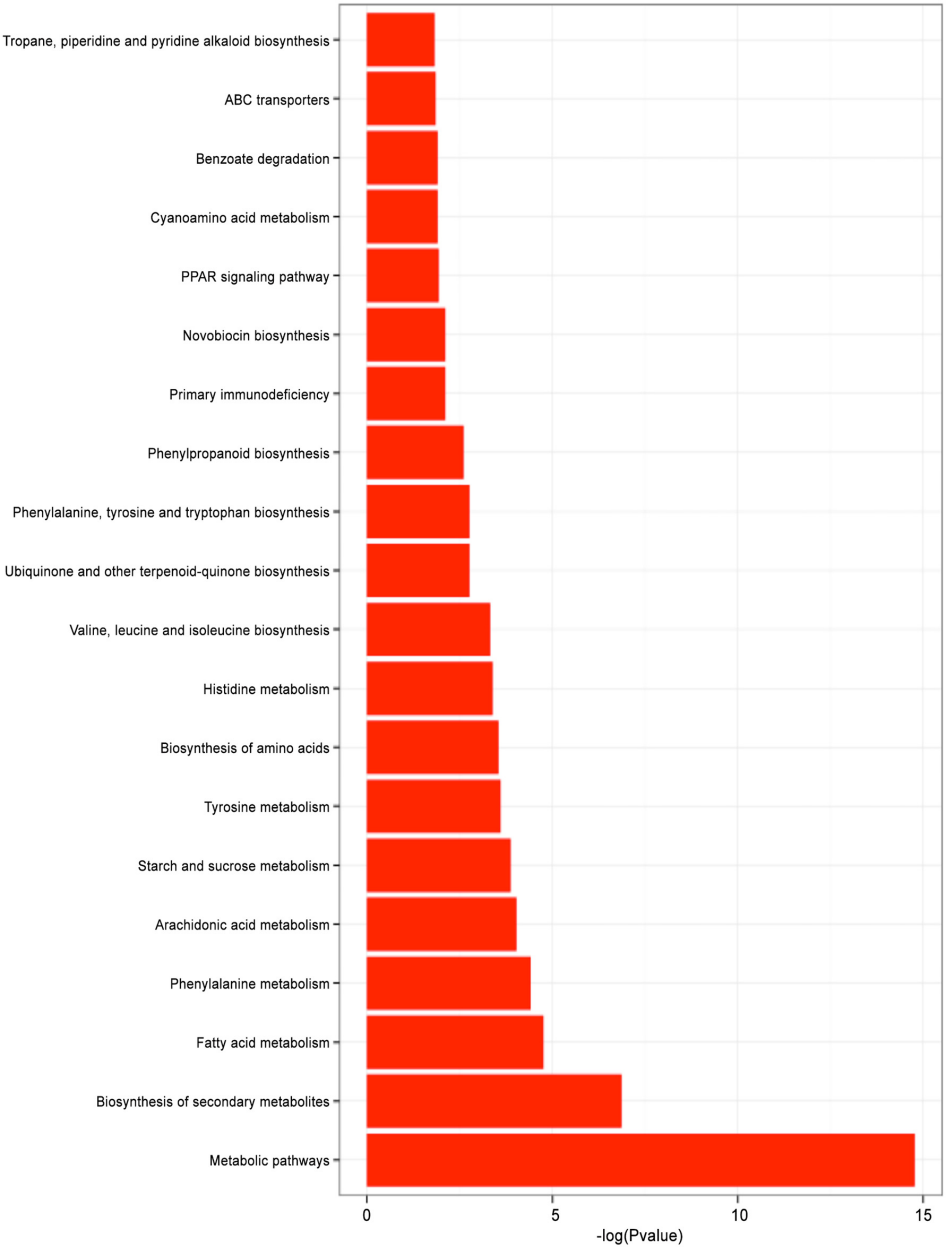
Histogram representation of top twenty most enriched KEGG pathways of DEGs in *S*. *parasitica* following copper sulfate treatment. Y-axis, KEGG pathway categories; x-axis, statistical significance of enrichment.

To further explore the DEG biological functions, we mapped DEGs to the KEGG database, and then enriched them to important pathways based on the whole transcriptome background. We mapped genes to 203 pathways. Many genes were found in multiple pathways; however, many genes were also restricted to a single pathway. There were 25 significantly enriched pathways, the most significantly enriched of which were metabolic pathways (Fig 5). The metabolism-related biological pathways included metabolic pathways, biosynthesis of secondary metabolites and biosynthesis of amino acids. A total of 98 genes were mapped to the metabolic pathways, 42 genes were mapped to the biosynthesis of secondary metabolites pathways, and 18 genes were mapped to the biosynthesis of amino acids. In addition, lipid metabolism-related biological pathways including fatty acid metabolism (13 genes), arachidonic acid metabolism (9 genes), and glycerolipid metabolism (6 genes), were also significantly enriched. Enriched amino acid metabolism-related biological pathways included phenylalanine metabolism (7 genes), tyrosine metabolism (7 genes), histidine metabolism (7 genes), valine, leucine and isoleucine biosynthesis (5 genes), and phenylalanine, tyrosine and tryptophan biosynthesis (5 genes).

The other significantly enriched pathways included carbohydrate metabolism-related starch and sucrose metabolic pathway (12 genes), metabolism of cofactors and vitamins related ubiquinone and other terpenoid-quinone biosynthesis pathway (4 genes), biosynthesis of other secondary metabolites related phenylpropanoid biosynthesis (4 genes), novobiocin biosynthesis (2 genes), tropane, piperidine and pyridine alkaloid biosynthesis (2 genes), immune diseases related primary immunodeficiency (4 genes), endocrine system related PPAR signaling pathway (8 genes), cyanoamino acid metabolism (3 genes), xenobiotic biodegradation and metabolism related benzoate degradation (3 genes), styrene degradation (2 genes), nitrotoluene degradation (2 genes), membrane transport related ABC transporters (11 genes),digestive system related protein digestion and absorption (5 genes), cancers overview related chemical carcinogenesis (3 genes).

In the metabolism-related biological pathways, the expression of certain genes (such as SPRG_05378, SPRG_01336, SPRG_00691, SPRG_19437, SPRG_17560, SPRG_13611, SPRG_08603, SPRG_09764, and SPRG_01617) were upregulated, while SPRG_11730, SPRG_04491, SPRG_10490, SPRG_12259, SPRG_04186, SPRG_18377, SPRG_18063, SPRG_06771, SPRG_16261, and SPRG_02541 genes were downregulated after copper sulfate treatment. These findings were consistent with the GO enrichment analysis, which indicated that copper sulfate mostly prevented the growth of S. *parasitica* by affecting protein synthesis and metabolism.

In the lipid metabolism-related biological pathways, among the 13 genes mapped to fatty acid metabolism, 12 genes were downregulated (including SPRG_15243, SPRG_02257, SPRG_06557, and SPRG_01190), but SPRG_09764. In the arachidonic acid metabolism, three genes were upregulated in S. *parasitica*, (SPRG_12960, SPRG_16543 and SPRG_13907), six genes were downreglated, (SPRG_20516, SPRG_10696, SPRG_09110, SPRG_11344, SPRG_18867 and SPRG_11345) after copper sulfate treatment. These results showed that copper sulfate affected S. *parasitica* by influencing the energy biogenesis deregulation.

Overall, the results of the DEGs pathway analysis support the viewpoint that copper sulfate inhibits *S*. *parasitica* growth by affecting multiple biological functions, such as energy biogenesis, protein synthesis and metabolism.

### Differential expression verification of differentially expressed genes

**Fig 6:**
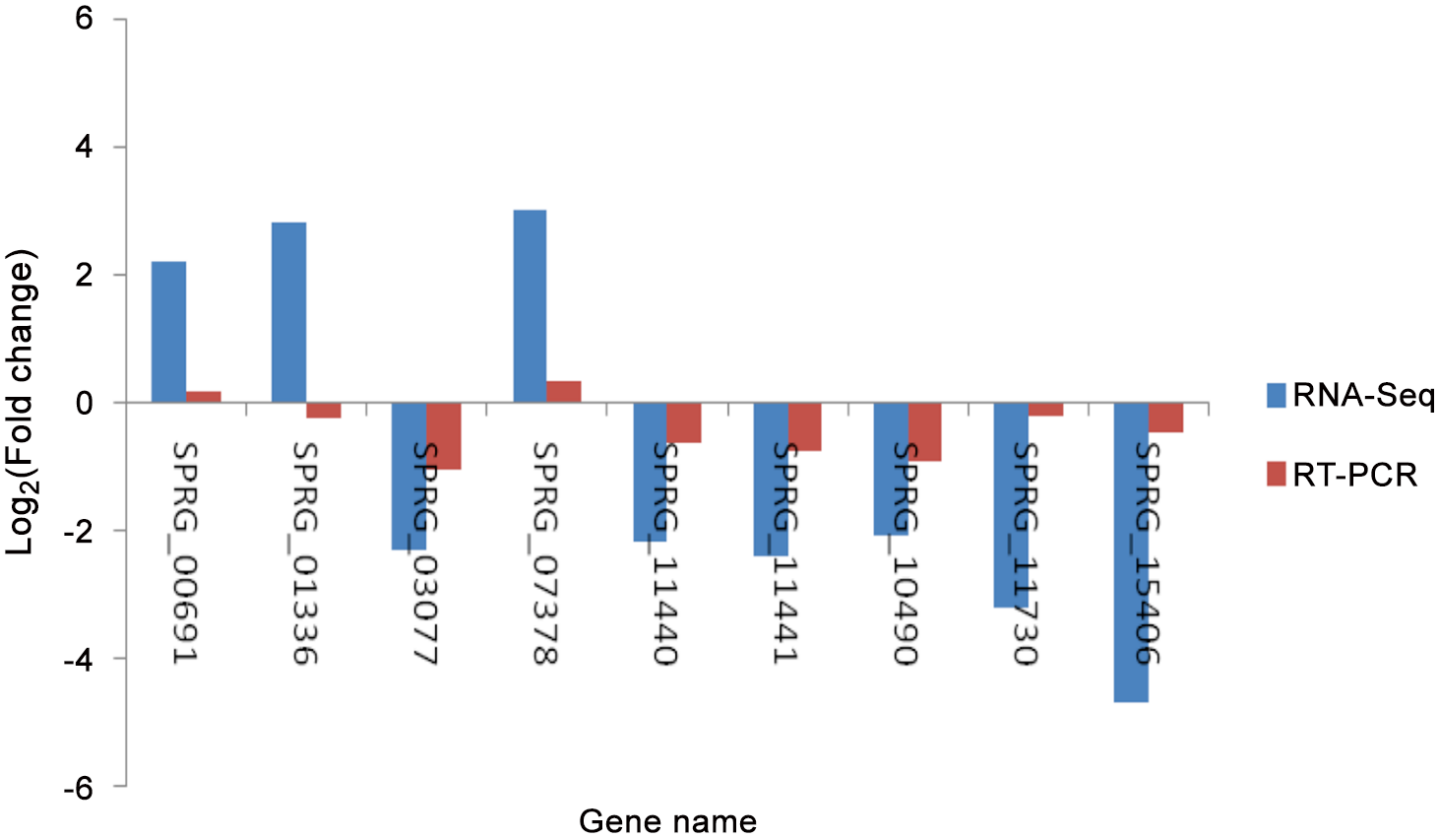
Comparison of nine genes expression levels between RNA-Seq and RT-PCR. Negative values indicate that the gene expression of *S*. *parasitica* was downregulated following copper sulfate treatment; positive values indicate that the gene expression was upregulated.

Nine genes with a clearly defined function were randomly selected from the differentially expressed genes identified by RNA-Seq to verify their expression using quantitative PCR. The primer sequences for all of the genes examined are listed in Table 3. The results verified that eight of the genes examined were consistent with the results of RNA-Seq (Fig 6). However, one gene (SPRG_01336), was not consistent with the RNA-Seq data. Overall, these results suggest that the RNA-Seq results are generally reliable; however, further studies are still needed to be carried out to verify the results from this study.

**Table 3:** Oligonucleotide primers of qRT-PCR for DEGs validation.

Gene name	Predict function	GO category	Pathway name	Forward primer (5’-3’)	Reverse primer (5’-3’)	Expected product
SpTub-b	-	-	-	AGACGGGTGCTGGTAACAAC	AGCGAGTGCGTAATCTGGAAA	136bp
SPRG_00691	Imidazoleglycerol-phosphate dehydratase	Carboxylic acid metabolic process (Biological process)	Metabolic pathways	GCGACGCTAACAACGACTGG	TTGTCGCCGTGCCCTGGTA	123bp
SPRG_01336	Maleylacetoacetate isomerase	Carboxylic acid metabolic process (Biological process)	Metabolic pathways	TGTAGAAGCGGGCGTTGA	CCGATGAGGCGGAAGAAG	178bp
SPRG_03077	TKL protein kinase	-	Primary immunodeficiency	GGGCAGCATCTTCTTCACAG	TCGCTTCAGAGTCAAGGGTC	195bp
SPRG_07378	Threonine ammonia-lyase	Carboxylic acid metabolic process (Biological process)	Metabolic pathways	CAACTGGGTCAAGCACTTTCG	GCAAAGAGCCCGACTTGGT	126bp
SPRG_11440	Uridine phosphorylase	Single-organism metabolic process (Biological process)	Metabolic pathways	TACAGCCTCTCGCACATTGG	CTTGGTGACTTCGTGGAGGAG	123bp
SPRG_11441	Uridine phosphorylase	Single-organism metabolic process (Biological process)	Metabolic pathways	CCACCTCGGTCTCTCGTACT	CTTGATCTCCAGCGTCTCGG	142bp
SPRG_10490	Ornithine-oxo-acid transaminase	Cofactor binding (Molecular function)	Metabolic pathways	GCCCGTGAAGACAAGTATGGT	CGTGAGTGCGTTCAAGATGC	174bp
SPRG_11730	Histidine ammonia-lyase	Carboxylic acid metabolic process (Biological process)	Metabolic pathways	CAAGCCGTCCGAACTCTTCA	GATCATCCACGGTCTTCTCAA	179bp
SPRG_15406	Trehalase	Hydrolase activity, acting on glycosyl bonds (Molecular function)	Metabolic pathways	TGGGCACAAAGCCATAGTCA	GATCATCCACGGTCTTCTCAA	98bp

## Discussion

Transcriptome sequencing is a powerful technique for studying the mechanism of changes of biological characteristics of an organism, and has been used successfully in some species [17–19]. In our study, we examined the mechanism by which copper sulfate inhibits the growth of S. *parasitica* by examining changes in the transcriptome after treatment using the Illumina sequencing platform. The total mapped rates of reads were 90.50% in the S. *parasitica* transcriptome of control group as compared to the reference genome, which met the required quality of the sequencing data needed for follow-up studies. The total unmapped reads was 9.50% of the total reads in the controls; however, the unmapped reads was 26.50% in the experimental group. It was remarkable that unmapped reads rate of the transcriptome of S. *parasitica* following treatment with copper sulfate was higher. This discrepancy might be due to the fact that the existing annotation is incomplete, or because copper sulfate caused gene variation.

The transcriptome of *S*. *parasitica* treated by copper sulfate contained more partial novel and complete novel splice junctions than that of control group. These findings indicate that copper sulfate may change the alternative splicing of certain genes that affect the metabolism and activity of *S*. *parasitica*. Therefore, investigating splice junctions may help understand the mechanism by which copper sulfate inhibits *S*. *parasitica*.

In addition to differences in splicing, copper sulfate may also affect *S*. *parasitica* by directly altering the expression of genes involved in key cellular processes. In order to investigate this, we classified DGEs into 1515 GO terms, consisting of three domains: biological process, cellular component, and molecular function. Of these, 262 GO terms were found to have dramatic expression changes. We mapped the DGEs to 203 pathways, with 25 of these pathways significantly enriched. The most significantly enriched pathways in this analysis were the metabolism-related biological pathways, including metabolic pathways, biosynthesis of secondary metabolites and biosynthesis of amino acids, which are responsible for the main biological functions of *S*. *parasitica*. Our study supplements the previous studies by Jiang *et al*., who investigated virulence genes in *S*. *parasitica* by sequencing its genome [20], and by Torto-Alalibo *et al*., who investigated expressed sequence tags and disclosed *S*. *parasitica* putative virulence factors [21].

In GO annotation enrichment, most of DGEs were related to protein synthesis and metabolism. However, in KEGG pathway analysis, the results showed not only substance metabolism was affected, but the production of energy was also affected. Together, these results indicate that copper sulfate inhibit *S*. *parasitica* growth by affecting multiple biological functions, which differ from the mechanisms of other anti–*S*. *parasitica* reagents. Such reagents include humic substances with higher molecular weights and aromaticity [7], clotrimazole [8], and saprolmycin A-E [9]. These data indicate that copper sulfate represents a novel anti–*S*. *parasitica* treatment.

We found that copper sulfate treatment did not alter the expression of the host-targeting protein 1 (SpHtp1) gene, which is responsible for *S*. *parasitica* translocation into host cells, suggesting that copper sulfate may not interfere with *S*. *parasitica*–host interactions [22].

Most of the genes in *S*. *parasitica* were hypothetical proteins, and the functions were not clear in the existing data. In order to facilitate further research, nine of the DGEs with clear functions were chosen randomly from the RNA-Seq data for qPCR to verify the results of the RNA-Seq. The qPCR results were essentially consistent with the results of the analysis of the transcriptome. One of the genes analyzed had a discrepant result, however, but we still believe the quality of the transcriptome of *S*. *para*sitica meet the requirements for studies on functional genes.

Taken together, copper sulfate represents a novel anti–*S*. *parasitica* agent that likely functions of inhibiting *S*. *parasitica* by affecting energy biogenesis, protein synthesis and metabolism. Our findings provide the basis for further investigation of the potential application of copper sulfate for controlling *S*. *parasitica* growth in aquaculture in the future.
